# Revenue-raising potential for universal health coverage in Benin, Mali, Mozambique and Togo

**DOI:** 10.2471/BLT.18.222638

**Published:** 2019-07-04

**Authors:** Inke Mathauer, Kira Koch, Samuel Zita, Alex Murray-Zmijewski, Mariam Traore, Nathalie Bitho, Nouria Brikci

**Affiliations:** a Department of Health Systems Governance and Financing, World Health Organization, Avenue Appia, 20, 1211 Geneva 27, Switzerland.; b Trade and Development Consultant, SZ Consulting, Maputo, Mozambique.; c Oxford Policy Management, Oxford, England.; d Programme National de Lutte contre la Tuberculose, Bamako, Mali.; e Oxford Policy Management, IIC Sarl., Lomé, Togo.

## Abstract

Increasing overall fiscal space is important for the health sector due to the centrality of public financing to make progress towards universal health coverage. One strategy is to mobilize additional government revenues through new taxes or increased tax rates on goods and services. We illustrate how countries can assess the feasibility and quantitative potential of different revenue-raising mechanisms. We review and synthesize the processes and results from country assessments in Benin, Mali, Mozambique and Togo. The studies analysed new taxes or increased taxes on airplane tickets, phone calls, alcoholic drinks, tourism services, financial transactions, lottery tickets, vehicles and the extractive industries. Study teams in each country assessed the feasibility of new revenue-raising mechanisms using six qualitative criteria. The quantitative potential of these mechanisms was estimated by defining different scenarios and setting assumptions. Consultations with stakeholders at the start of the process served to select the revenue-raising mechanisms to study and later to discuss findings and options. Exploring feasibility was essential, as this helped rule out options that appeared promising from the quantitative assessment. Stakeholders rated stability and sustainability positive for most mechanisms, but political feasibility was a key issue throughout. The estimated additional revenues through new revenue-raising mechanisms ranged from 0.47–1.62% as a share of general government expenditure in the four countries. Overall, the revenue raised through these mechanisms was small. Countries are advised to consider multiple strategies to expand fiscal space for health.

## Résumé

Il est important d'accroître l’espace budgétaire global alloué à la santé en raison du caractère crucial du financement public pour accomplir des progrès en faveur de la couverture sanitaire universelle. Une stratégie consiste à mobiliser des fonds publics supplémentaires par le biais de nouvelles taxes ou d'une augmentation des taux d'imposition applicables aux biens et aux services. Nous expliquons comment les pays peuvent évaluer la faisabilité et le potentiel quantitatif de différents mécanismes de mobilisation de fonds. Nous examinons et synthétisons les processus et les résultats d'évaluations nationales menées au Bénin, au Mali, au Mozambique et au Togo. Ces études ont analysé la mise en place de nouvelles taxes ou la hausse de taxes sur les billets d'avion, les appels téléphoniques, les boissons alcoolisées, les services touristiques, les transactions financières, les billets de loterie, les véhicules et les industries extractives. Les équipes chargées des études au sein de chaque pays ont évalué la faisabilité des nouveaux mécanismes de mobilisation de fonds à l'aide de six critères qualitatifs. Le potentiel quantitatif de ces mécanismes a été estimé en définissant différents scénarios et en formulant des hypothèses. Des consultations ont été menées auprès des parties prenantes au début du processus afin de sélectionner les mécanismes de mobilisation de fonds à étudier et de discuter des résultats et des options à un stade ultérieur. Il était essentiel d'étudier la faisabilité, car cela a permis d'écarter les options qui semblaient prometteuses à partir de l'évaluation quantitative. Les parties prenantes ont jugé la stabilité et la durabilité positives pour la plupart des mécanismes, mais la faisabilité politique a été une question clef tout au long du processus. Nous avons estimé que la part des fonds supplémentaires générés par les nouveaux mécanismes de mobilisation de fonds dans les dépenses générales de l'État allait de 0,47 à 1,67% dans les quatre pays. Dans l'ensemble, les fonds générés par ces mécanismes étaient de faible ampleur. Il est conseillé aux pays d'envisager plusieurs stratégies pour augmenter l’espace budgétaire alloué à la santé.

## Resumen

El aumento del espacio fiscal general es importante para el sector de la salud debido al carácter central de la financiación pública para avanzar hacia una cobertura sanitaria universal. Una estrategia consiste en movilizar fondos públicos adicionales mediante nuevos impuestos o aumentar los tipos impositivos sobre los bienes y servicios. A continuación se ilustra cómo los países pueden evaluar la viabilidad y el potencial cuantitativo de los diferentes mecanismos de recaudación de fondos. Se han revisado y sintetizado los procesos y los resultados de las evaluaciones nacionales en Benin, Malí, Mozambique y Togo. Los estudios analizaron nuevos impuestos o la subida de los impuestos sobre los billetes de avión, las llamadas telefónicas, las bebidas alcohólicas, los servicios turísticos, las transacciones financieras, los billetes de lotería, los vehículos y las industrias de extracción. Los equipos de estudio de cada país evaluaron la viabilidad de nuevos mecanismos de recaudación de fondos mediante seis criterios cualitativos. El potencial cuantitativo de estos mecanismos se estimó mediante la definición de diferentes escenarios y el establecimiento de supuestos. Las consultas con las partes interesadas al comienzo del proceso sirvieron para seleccionar los mecanismos de recaudación de fondos que se estudiarían y posteriormente examinar las conclusiones y las opciones. Era esencial explorar la viabilidad, ya que ayudaba a descartar opciones que parecían prometedoras de la evaluación cuantitativa. Las partes interesadas calificaron la estabilidad y la sostenibilidad como positivas para la mayoría de los mecanismos, pero la viabilidad política fue una cuestión clave en todo momento. Los fondos adicionales estimados por medio de los nuevos mecanismos de recaudación oscilaron entre el 0,47 % y el 1,62 % de los gastos de las administraciones públicas de los cuatro países. En general, los fondos recaudados mediante estos mecanismos fueron reducidos. Se aconseja a los países que consideren múltiples estrategias para ampliar el espacio fiscal para la salud.

## ملخص

إن زيادة الحيز المالي العام أمر هام بالنسبة لقطاع الصحة وذلك بسبب مركزية التمويل العام لإحراز تقدم تجاه تغطية صحية شاملة. تتمثل إحدى الاستراتيجيات في تحقيق إيرادات حكومية إضافية من خلال فرض ضرائب جديدة، أو زيادة معدلات الضرائب على السلع والخدمات. نحن نوضح كيف يمكن للدول تقييم الجدوى والإمكانات النوعية للآليات المختلفة لجمع الإيرادات. كما نقوم باستعراض وتقنين العمليات والنتائج من تقييمات الدولة في بنن ومالي وموزامبيق وتوجو. قامت الدراسات بتحليل الضرائب الجديدة أو الضرائب المرفوعة على تذاكر الطيران، والمكالمات الهاتفية، والمشروبات الكحولية، والخدمات السياحية، والمعاملات المالية، وتذاكر اليانصيب، والمركبات، والصناعات الاستخراجية. قامت فرق الدراسة في كل دولة بتقييم مدى جدوى الآليات الجديدة لزيادة الإيرادات، باستخدام ستة معايير نوعية. تم تقدير الإمكانات النوعية لهذه الآليات عن طريق تحديد سيناريوهات ووضع افتراضات مختلفة. أدت الاستشارات مع أصحاب المصلحة في بداية العملية لاختيار آليات جمع الإيرادات للدراسة، ومناقشة النتائج والخيارات لاحقاً. كان استكشاف الجدوى أمراً ضرورياً، حيث ساعد ذلك في استبعاد الخيارات التي بدت واعدة نتيجة للتقييم النوعي. قام أصحاب المصلحة بتقييم الاستقرار والاستدامة بكونها إيجابية بالنسبة لمعظم الآليات، إلا أن الجدوى السياسية كانت جانباً بارزاً طوال الوقت. تراوحت الإيرادات الإضافية المقدرة من خلال آليات جمع الإيرادات الجديدة، من 0.47 إلى 1.62٪ كحصة من الإنفاق الحكومي العام في الدول الأربع. بشكل عام، كانت الإيرادات التي تم جمعها من خلال هذه الآليات، قليلة. ويتم نصح هذه الدول بالتفكير في استراتيجيات متعددة للتوسع في الحيز المالي للصحة.

## 摘要

由于公共融资在实现全民健康覆盖上的重要性,因此增加总体财政空间对卫生部门至关重要。策略之一是通过对商品和服务开征新税或提高税率,从而提高政府的额外收入。我们旨在论证各国如何评估不同收益机制的可行性和定量潜力。我们评审并综合了贝宁、多哥、马里和莫桑比克的评估进程和结果。这些研究就在机票、电话、酒精饮料、旅游服务、金融交易、彩票、汽车业和采掘业方面开征新税和提高税率进行了分析。各国的研究小组使用了 6 个定性标准来评估新收益机制的可行性。这些机制的定量潜力是通过定义不同的情景和设定假设来估计的。进程开始时,与利益相关者协商有助于选择要研究的收益机制,并随后讨论调查结果和备选方案。探索可行性是必要的,这有助于从定量评估中排除看似可行的方案。利益相关者对大多数机制的稳定性和可持续性给予了正面评价,但政治上的可行性自始至终都是问题的关键。在这四个国家,通过新收益机制获得的额外收入约占一般性政府财政支出的 0.47%-1.62%。总而言之,通过此类机制获得的增收依然有限。建议各国考虑多项策略来扩大卫生财政空间。

## Резюме

Расширение фискальной сферы в целом важно для здравоохранения, так как централизованный характер общественного финансирования помогает добиваться целей, связанных со всеобщим охватом медицинскими услугами. Одной из стратегий является привлечение дополнительных источников госдохода путем введения новых налогов или увеличения ставок налогообложения товаров и услуг. Авторы на примере показывают, как страны могут оценить осуществимость и количественный потенциал различных механизмов повышения дохода. Авторы изучили и обобщили процессы и результаты, полученные в ходе оценки таких стран, как Бенин, Мали, Мозамбик и Того. В ходе исследований были проанализированы новые налоги или повышение налогов на авиабилеты, мобильную связь, алкогольные напитки, туристические услуги, финансовые транзакции, лотерейные билеты, автомобили и продукцию добывающей промышленности. Группы исследователей в каждой из стран оценили осуществимость новых механизмов повышения дохода с помощью шести качественных критериев. Количественный потенциал этих механизмов оценивался с использованием различных сценариев и вариантов регулирования. Консультации с партнерами в начале процесса помогли выбрать изучаемые механизмы и обсудить результаты и возможности. Изучение осуществимости таких механизмов имело критически важное значение, поскольку помогло исключить варианты, которые казались многообещающими с точки зрения количественной оценки. Партнеры положительно оценили стабильность и возможность устойчивого развития для большинства механизмов, но во всех случаях ключевым фактором оказывалась политическая осуществимость. Оценка прироста доходов за счет новых механизмов их повышения оказалась в пределах 0,47–1,62% доли общих правительственных расходов в четырех странах. В целом прирост дохода за счет таких мероприятий был малым. Странам рекомендовано рассмотреть несколько различных стратегий расширения фискальной сферы для поддержки здравоохранения.

## Introduction

Countries may need to raise additional funds to progress towards universal health coverage (UHC). This implies increasing the fiscal space for health. Fiscal space has been defined as “the ability of governments to increase spending for the sector without jeopardizing the government’s long-term solvency or crowding out expenditure in other sectors needed to achieve other development objectives.”^1^

Fiscal space for health can be expanded in several ways: general economic growth in a country; increased state or tax revenues and improved tax collection; an increased proportion of government spending on health; and improved efficiency in the use of funds.^1^^,^^2^ Mobilizing additional tax revenues can be done by introducing new taxes or increasing existing tax levels. Imposing taxes on specific products and services to increase general government revenue has also gained attention through the World Health Report 2010.^3^ Countries’ interest in resource expansion for health is increasingly important in the light of decreasing levels of funding by global health initiatives to low- and middle-income countries.^4^ Importantly, raising additional revenue for health needs to be examined within the context of overall government revenues, of which health is only one component. The objective to increase fiscal space for health does not necessarily require new revenues to be earmarked for the health sector, although some countries do so. Instead, the aim is to increase overall government revenues and augment the share going to health.^2^

While a mix of strategies may be needed to expand fiscal space, we focus in this paper on mechanisms for raising additional government revenue. We illustrate how countries can assess the feasibility and quantitative potential of the mechanisms. To do this, we review and synthesize such processes and results from four country studies in Benin, Mali, Mozambique and Togo.^5^^–^^8^ The studies were part of the countries’ efforts to develop strategies to expand UHC.

## Context of country studies

Table 1 summarizes key demographic, health and health coverage indicators of the four countries. The data show that there is still a long way to go towards UHC. For example, the UHC service index which measures coverage of essential health services ranged from 32 to 42 across the four countries, compared with above 70 in Organisation for Economic Co-operation and Development countries.^11^

**Table 1:** Key demographic, health and health coverage indicators in Benin, Mali, Mozambique and Togo

Variable	Benin	Mali	Mozambique	Togo
Population in thousands^9^	10 872	17 995	28 830	7 606
% of population in the informal economy (year)^10^	95 (2011)	93 (2015)	NA	93 (2011)
Maternal mortality ratio^a^ in 2015^11^	405	587	489	368
Under-five mortality rate^b^ in 2017^11^	98	106	72	73
% of 1-year-olds receiving DTP3 in 2017^11^	82	66	80	90
No. of medical doctors per 10 000 people in 2009–2018^11^	1.6	1.4	0.7	0.5
% of population with catastrophic health expenditure^c^ (year of latest available data)^12^	11.11 (2003)	3.38 (2006)	1.19 (2008)	10.65 (2006)
% of births with skilled health personnel in 2009–2018^11^	78	44	73	45
UHC service coverage index^d^ in 2015^11^	41	32	42	42

DTP3: third dose of diphtheria, tetanus and pertussis vaccine; NA: not available; UHC: universal health care.

^a^ The maternal mortality ratio is the number of maternal deaths per 100 000 live births.

^b^ The number of deaths of infants and children under five years of age per 1000 live births.

^c^ Percentage of the population with household expenditure on health exceeding 10% of total household expenditure or income.

^d^ The universal health coverage service coverage index (range 0–100) is a measure of sustainable development goal indicator 3.8.1, which is coverage of essential health services (defined as the average coverage of essential services based on tracer interventions that include reproductive, maternal, newborn and child health, infectious diseases, noncommunicable diseases and service capacity and access, among the general population, and the most disadvantaged groups).

The share of the population working in the informal sector is high (Table 1). Currently, people rely largely on underfunded, government health services. Benin has begun to build up a national insurance scheme in which funds from the government budget would be used to finance the health coverage of the very poorest people and to partially subsidize poor people, while higher economic groups would make contributions.^13^ In Mali, the parliament approved a law in 2018 on a national universal health insurance scheme, but implementation has not yet started. The idea is to use state budget transfers to subsidize the contributions of vulnerable and poor population groups in the informal economy. The health ministry has projected the funds needed to provide these subsidies, with a core assumption being an increased budget for the health sector.^14^ However, the precise source of revenue and which additional revenue-raising mechanisms will be applied has not yet been decided. In Togo, the health ministry is in the process of finalizing a national health financing strategy. The existing mandatory health insurance scheme is still limited to current and retired civil servants and their family members, and covers 4% of the population in 2019.^15^ Contributions are paid by the civil servants and their employer (government agencies). Hence, a core question is how to expand coverage to the whole population. Technical debates currently focus around the idea of using budget transfers to cover people in the informal economy.^16^ Benin, Mali and Togo are members of the West African Economic and Monetary Community. The Community provides a harmonized tax framework, which sets a limit on specific taxes (tobacco products and alcoholic drinks, for instance) and has harmonized taxation rules for certain sectors, such as banking and aviation.^17^

In Mozambique, the government has developed a health financing strategy, which is currently subject to approval from ministries. In this strategy, the aim is to define various mechanisms to raise financial resources to enhance fiscal space.^18^ Mozambique is part of the Southern African Development Community, which also seeks to harmonize certain tax rates among member countries.^19^

Table 2 presents some key health expenditure indicators and reveals that domestic general government health expenditure as a share of current health expenditure is low in the three west African countries (ranging from 20.0% to 31.1 %). In Mozambique, the figure is higher (53.3%), but its per capita current health expenditure is also much lower than in the other three countries. The priority given to health and hence the budget allocation to health (which includes domestic general government health expenditure and the external funds flowing into the health budget) as a share of general government expenditure is still rather low.^20^ Likewise, general government expenditure as a share of gross domestic product (GDP) is still low for Benin and Mali (21.3% and 22.2%, respectively), compared with 31% and 41% in upper-middle- and high-income countries.^21^ Global cross-country evidence shows that the absolute level of public spending matters and a systematic improvement in UHC performance, in particular a lower incidence of catastrophic health expenditure, is observed when public spending on health increases.^22^^,^^23^ Thus, the four countries’ UHC expansion efforts would benefit from more revenues through an overall increased government budget and a higher share of this going to health.

**Table 2:** Health expenditure indicators for 2016 (latest data available) in Benin, Mali, Mozambique and Togo

Variable	Benin	Mali	Mozambique	Togo
GDP per capita, US$	788	780	379	586
Current health expenditure per capita, US$	30	30	19	39
General government expenditure as a share of GDP, %	21.3	22.2	32.4	31.2
Current health expenditure as a share of GDP, %	3.9	3.8	5.1	6.6
Domestic general government health expenditure as a share of general government expenditure, %	3.7	5.3	8.3	4.3
Domestic general government health expenditure as a share of GDP, %	0.8	1.2	2.7	1.3
Domestic general government health expenditure as a share of current health expenditure, %	20.5	31.1	53.3	20.0
External health expenditure as a share of current health expenditure, %	30.5	32.7	38.1	20.7
Out-of-pocket expenditure on health as a share of current health expenditure, %	43.5	35.3	7.7	50.4

GDP: gross domestic product; US$; United States dollars.

Note: County populations are shown on Table 1.

Source: Based on World Health Organization global health expenditure database.^9^

## Illustrating the assessment approach

We outline a four-step method and process that was applied to assess new revenue-raising mechanisms in the four country studies. Each country study was part of the technical and policy advisory support process that was requested from the World Health Organization (WHO). Each country study team consisted of a national and international consultant, from among the authors with this specific expertise, accompanied by the country’s health ministry and WHO country office and headquarters staff.

### Multistakeholder consultation

The first step was a multistakeholder consultation in each country that served to pre-select the new revenue-raising mechanisms to be explored in detail. A wide range of stakeholders participated in a one-day meeting: representatives from ministries of health, finance, tourism services and infrastructure; civil society organizations; development partners; and the private sector. Following the same format and approach in each country, study teams presented a range of revenue-raising mechanisms, with their advantages and disadvantages, based on evidence from the literature. Small group and final plenary discussions of what stakeholders considered useful resulted in a shortlist. The list was screened for a final selection of four to five revenue-raising mechanisms to be explored in depth (Box 1).

Box 1 **Revenue-raising options discussed at stakeholder consultations and selected for the in-depth analysis in the country studied**

**Benin**

Discussion of taxes on: airplane tickets, financial transactions, alcoholic drinks, tobacco products, public contracts, imported vehicles, petroleum products, extractive industries, large companies, real estate property, luxury products, companies with large volume of pollution, household garbage, mobile phones, large cars, lotteries and gambling, health insurance contracts, pharmaceutical companies of branded medicines, voluntary diaspora contributions, or an increase of VAT and of traffic violation fees.

Selected taxes for in-depth analysis on: alcoholic drinks, airplane tickets, telephone (mobile), financial transactions and national lottery.

**Mali**

Discussion of taxes on: airplane tickets, visa applications, alcoholic drinks, tobacco products, public contracts, hydrocarbon, hotel nights, extractive industries, sugar-sweetened drinks, real estate property, transport companies, companies with large volume of pollution, earnings of ministers and deputies, mobile phone calls, livestock exports, lotteries and gambling, pharmaceutical companies of branded medicines, voluntary diaspora contributions, road tolls, financial transactions; or an increase of municipal taxes and of VAT.

Selected taxes for in-depth analysis on: alcoholic drinks, airplane tickets, telephone (mobile and fixed), financial transactions and extractive industries.

**Mozambique**

Discussion of taxes on: alcoholic drinks, tourism services, vehicles, extractive industries, private clinics, forestry and wildlife activities.

Selected taxes for in-depth analysis on: alcoholic drinks, tourism services, vehicles and extractive industries.

**Togo**

Discussion of taxes on: airplane tickets, financial transactions, alcoholic drinks, tobacco products, public contracts, imported vehicles, petroleum products, extractive industries, large companies, real estate property, luxury products, companies with large volume of pollution, household garbage, mobile phones, large cars, lotteries and gambling, health insurance contracts, pharmaceutical companies of branded medicines, voluntary diaspora contributions, or an increase of VAT and of traffic violation fees.

Selected taxes for in-depth analysis on: alcoholic drinks, airplane tickets, telephone (mobile and fixed), financial transactions and extractive industries.

VAT: value-added tax.Source: Based on country studies.^5^^–^^8^

### Feasibility analysis

In the second step, each country team conducted a detailed qualitative analysis of the feasibility of the selected mechanisms. This started with a literature and document review, which informed the subsequent data collection process. A series of semi-structured interviews were held with key stakeholders from government agencies, the private sector and development partners. The interviews provided insights into current taxation mechanisms and rates in the respective sectors, the feasibility of the mechanisms explored, and potential challenges, such as whether stakeholders would support or resist the introduction of a new revenue-raising mechanism. This qualitative analysis was guided by six criteria looking at various aspects of feasibility (Box 2). The criteria were developed during the first country study in Togo^5^ and applied in the other three studies. We graded the criteria from very weak to very strong based on the data from stakeholders’ discussions and interviews.

Box 2 **Feasibility criteria and related key questions for the qualitative assessment of revenue-raising mechanisms**

**Political feasibility**

Is there political will for this funding mechanism, or does it create reluctance at the political level (whether from government or civil society)?

**Sustainability**

Would the mechanism be applicable in the long term?

**Stability**

Would revenues be stable over time?

**Progressivity (equity in financing)**

Would financially better-off people likely contribute with a larger proportion of their income than poorer people?

**Administrative efficiency**

Are institutional and operational arrangements in place to implement the financing mechanism? What would be the risks of fraud and corruption and how could these be reduced?

**Other possible effects**

Which (positive or negative) effects would this revenue-raising mechanism have on the supply and demand of particular goods and services?

Source: Adapted from Brikci & Bitho, 2014.^5^

### Quantitative analysis

The third step was the quantitative analysis. The country teams collected data from country statistics and global databases, such as World Bank development indicators, the International Monetary Fund’s world economic outlook indicators and WHO global health expenditure data. This step also served to set assumptions and projection variables to estimate potential revenues for different scenarios, for a defined projection period which was determined at the stakeholder meetings. Box 3 illustrates the approach to estimating revenues, taking the example of a tax on airplane tickets in Togo.

Box 3 **Example of scenario definitions and assumptions set to estimate revenues from an airplane ticket tax in Togo**

Projection period: 10 years

Definition of different taxation scenarios:

- scenario 1: taxing only passengers going abroad; distinction of taxes between economy class and business class;
- scenario 2: scenario above plus taxing arrival passengers;
- scenario 3: scenario 2 plus taxing transit passengers.

Setting of assumptions over the projection period for: economic growth, demand elasticity and inflation rates; share of business-class or first-class versus economy-class passengers.

Projection of the number of passengers departing from, in transit and arriving in the country, in business and economy class, over the projection period, based on the above assumptions.

Calculation of potential revenues, using the above scenarios and assumptions, was done using the following formula:

revenues (in national currency) = tax rate (%) x tax base (in national currency)

with the tax base calculated as: number of services or number of consumed products multiplied by the elasticity factor, projected over the number of years with estimated growth rate and inflation rate for each year.

Note: Explanations on more detailed formulas can be found in country studies ^5^^–^^8^ and Vigo & Lauer, 2017.^24^Source: Adapted from Brikci & Bitho, 2014.^5^

Different high and low scenarios were specified for each mechanism to estimate potential revenues for the defined period (Table 3). For example, a high scenario was based on a higher tax rate or assumptions of higher increases in the consumption of a product or a higher growth rate over the projection period.

**Table 3:** Illustrations of low- and high-scenario settings for each revenue-raising mechanism in Benin, Mali, Mozambique and Togo

Country and tax to be considered	Low scenario	High scenario^a^	Options proposed for consideration
**Benin**			
Alcoholic drinks	NA	Increase in tax rate by 15%; currently 15% on beers and ciders; 35% on wine; 40% on spirits & champagne^a^	High scenario
Airplane tickets	NA	New levy of US$ 20 on airplane tickets	High scenario
Telephone (mobile)	NA	New tax of 2% on airtime or mobile phone credits	High scenario
Financial transactions	NA	New tax of 5% on official remittances	NA
National lottery	NA	New tax of FCFA 200 per ticket, based on the average price of a lottery ticket	High scenario
**Mali**			
Alcoholic drinks	Increase in tax rate by 5% on imported alcoholic drinks	Increase in tax rate by 15% on imported alcoholic drinks	High scenario
Airplane tickets	Increased taxes on tickets for passengers going abroad: economic class FCFA 15, business class FCFA 150; arriving: FCFA 15; in transit: FCFA 15	Increased taxes on tickets for passengers going abroad: economic class FCFA 25, business class FCFA 250; arriving: FCFA 150; in transit: FCFA 25	High scenario
Telephone (mobile and fixed)	New tax of 1% tax on operators’ revenues	New tax of 3% on operators’ revenues	New tax of 2% on operators’ revenues
Financial transactions	New tax of 0.01% on diaspora remittances	New tax of 1% on diaspora remittances	NA
Extractive industries	No scenarios defined^b^	No scenarios defined^b^	NA
**Mozambique**			
Alcoholic drinks	New tax of 1% on retail price of beer, 2% on wine and 5% on spirits	New tax of 1% on retail price of beer, 2% on wine and 10% on spirits	Low scenario
Tourism services	New tax of 1% on cost of accommodation	Same as low scenario^c^	Low scenario
Vehicles, cars	Increase in statutory tax rates by 10% once every 3 years	Increase in statutory tax rates by 20% once every 3 years	Low scenario
Extractive industries	10% minimum statutory rate of hypothecation; annual growth rate of tax revenues equal to a minimum of 5% (earmarking)	10% minimum statutory rate of hypothecation; annual growth rate of tax revenues equal to a minimum of 15% (earmarking)	Low scenario
**Togo**			
Alcoholic drinks	Increase in tax rate by 15% on all imported alcoholic drinks	Increase in tax rate by 10% on beer from the local brewery, and a 15% increase in the tax on all imported alcoholic drinks	High scenario
Airplane tickets	Increased taxes on tickets for passengers going abroad: economy class FCFA 10, business class FCFA 100; arriving: FCFA 10; in transit: FCFA 10	Increased taxes on tickets for passengers going abroad: economy class FCFA 20, business class FCFA 200; arriving: FCFA 30; in transit: FCFA 20	High scenario
Telephone (mobile and fixed)	New tax on calls of 1 FCFA per minute	New tax on calls of 5 FCFA per minute	Low scenario
Financial transactions	New tax of 0.01% on diaspora remittances	New tax of 1% on diaspora remittances	NA
Extractive industries	No scenarios defined^b^	No scenarios defined^b^	NA

FCFA: West African CFA franc; NA: not assessed and/or not proposed for consideration; US$: United States dollars.

^a^ No data on alcoholic drinks taxes, prices and consumption were available in Benin. Instead, average revenues of other countries were used as an approximation. West African Economic and Monetary Union tax ceiling of alcoholic drinks beverages of 50% needed to be considered.

^b^ No scenario defined due to lack of data

^c^ Due to lack of accurate data and simplicity, it was assumed that circumstances would remain the same as under the low scenario.

Notes:  A new tax refers to introducing a new type of tax, independent of whether another type of tax (for example a value added tax) existed on the same product or service. An increased tax rate refers to an existing tax that is raised.

Source: Based on country studies.^5^^–^^8^

### Stakeholder feedback discussions

In the fourth and last step of this process the country teams reported back the results of the qualitative and quantitative analysis to all stakeholders and decision-makers at a workshop to receive feedback on the suggestions. The workshop also served to build ownership on the conclusions and translate the analysis into an agreed way forward for policy discussions and decisions on next practical steps, also in relation to the development or the implementation of the health financing strategy.

## Illustrations of country findings

The list of mechanisms selected for the in-depth studies and the feasibility issues expressed by stakeholders were similar in the three West African countries (Table 4). Stability and sustainability were rated positive for most mechanisms, except for a tax on the extractive industries and national lottery tickets. Stakeholders thought that a new tax on remittances might raise equity concerns due to potentially negative impacts on lower income groups. Tax differentiations between consumer goods (wines and spirits versus beer in the case of a tax on alcoholic drinks) and consumer groups (business versus economy passengers in the case of a tax on airplane tickets) can make the tax more progressive. Political feasibility seemed to be an issue for nearly all the mechanisms assessed. Taking all feasibility criteria into consideration, new taxes or increased tax levels on alcoholic drinks, airplane tickets and telephone calls received the most positive ratings in the feasibility assessment. Taxes on national lottery tickets, financial transactions and the extractive industries were rated as less acceptable. Stakeholders argued that the financial sector and extractive industries are emerging and need to attract investors and the political situation around the extractive industries was still unclear.

**Table 4:** Illustrations of feasibility considerations on revenue-raising mechanisms in Benin, Mali and Togo

Criterion	Increased tax on (imported) alcoholic drinks	New^a^ or increased^b^ tax on airplane tickets	New tax on telephone communications	New tax on remittances in financial transactions	New tax on the extractive industries^c^	New tax on national lottery tickets^d^
Political feasibility	Resistance from the population would be expected, especially for a tax on beer (–)	Unitaid airline tax was previously rejected by parliament in Togo but is already in force in Mali. Tax for the purpose of UHC may gain more acceptance (+ –)	Competing interests of ministries (–)	Resistance from the population would be expected (–)	Competing interests of ministries. Unclear political situation (– –)	Popularity of gambling may be an advantage to advocate for UHC. Tax on national lottery tickets already exists to fund social, cultural and sport events (+)
Sustainability	No high consumption rates so far, but increase would be expected (+)	Growing industry (+ +)	Growing industry (+ +)	Growing amount of remittance from migrants (+)	Growing industry (+)	Revenues may be unreliable due to irregular consumers (–)
Stability	Stable market (+)	Stable market (+)	Stable market (+)	Stable market (+)	Revenues would fluctuate due to varying commodity prices (–)	No stable market (–)
Progressivity	Taxes could be higher for wine and spirits which are consumed by more affluent population groups (compared with beer) to be more progressive (+ +)	Affects more affluent population groups. Distinction between economic and business class passengers would enhance progressivity (+ +)	A flat tax rate is more progressive. The tax would be more progressive if differentiated in terms of volume and services (+ –)	Potential negative impact for people who depend on remittances, as those who receive remittances spend the highest proportion of their income on consumption (– –)	Not enough information to assess this	Potential negative impact for low-income groups (–)
Administrative efficiency	Mechanism to collect taxes already in place (+ +)	Mechanism to collect taxes already in place (+ +)	Mechanism to collect taxes already in place (+ +)	No information available	No effective collection mechanism in place. Lack of data on how much is collected (Togo) (– –)	Mechanism to collect taxes already in place (++)
Other possible effects and trade-offs	Has the potential to reduce alcoholic drinks consumption, which increases health status of the population (+)	Marginal risk that national airports would lose competitiveness (+ –)	Investments may slow down, which would affect the rural poor who depend on telephone services (–)	Informal transactions would benefit. (– –)	Extractive industries already highly taxed (Mali). This emerging sector still needs to attract investors (– –)	Current market is competitive, with diverse gambling options. Existing lottery already in place (–)

UHC: universal health coverage.

^a^ Only in Benin and Togo.

^b^ Only in Mali.

^c^ Only in Mali and Togo.

^d^ Only in Benin.

Note: We graded the criteria from very weak to very strong based on the data from stakeholders’ discussions and interviews: (– –) = very weak; (–) = rather weak; (+–) = neutral; (+) = strong; (+ +) = very strong.

Sources: Based on country studies.^5^^–^^7^

For Mozambique, stakeholders assessed most of the studied mechanisms positively regarding sustainability, progressivity and potential trade-offs, but rated political feasibility lower, due to the likely competing interests of different ministries (Table 5). Moreover, administrative efficiency was a concern for taxes on the extractive industries, since the set-up and running costs of the tax are expected to be high and technical capacity to be weak. Overall, stakeholders rated new taxes on alcoholic drinks and on tourism services as more promising.

**Table 5:** Illustrations of feasibility considerations on revenue-raising mechanisms in Mozambique

Variable	New tax on alcoholic drinks	New tax on tourism services	Increased tax on vehicles	Earmarking of a share of revenues from the extractive industries
Political feasibility	Competing interests among ministries. Local producers may claim high sector-specific taxes already exist (–)	Competing interests among ministries (–)	Competing interests among ministries. Revision of law could be complex and lengthy. Autonomy of municipalities might create friction with the central ministry if earmarked (or lead to eventual delays of transferring funds) (–)	Competing interests among ministries (–)
Sustainability	Levy needs to be high enough to deter abusive alcohol consumption or to represent a good source of revenue (+)	A 1–3% levy would probably not provoke shifts in the demand for different types of tourist accommodation (+)	Price elasticity of demand for cars is fairly rigid. No effective and efficient alternative means of (public) transport is in place (+)	Already annually collected and in place for the lifetime of natural resources (+)
Stability	Growing industry (+)	Growing industry and competitive environment (+)	No major fluctuations, at least for light and heavy vehicles in the short and medium term (+)	Revenues depend on fluctuations of international commodity prices, but industries overall are growing (+ –)
Progressivity	With a high level of current smuggling, the burden of a new levy would likely affect the formal sector (+ –)	The burden of the levy would increase with the price of accommodation (+ +)	The levy would be mostly incurred by vehicle owners who can afford to purchase and maintain a vehicle (+)	The tax burden of different income groups would not be affected through this earmarking
Administrative efficiency	Mechanisms to collect taxes are already in place (++)	No information available	Running costs would be high. Building technical capacity will be crucial (– –)	No mechanisms are in place. Running costs would be high. Inter-ministerial management committee is required (– –)
Other possible effects and trade-offs	Potential to reduce alcohol consumption, which increases the health status of the population (+)	Supply side will likely be challenged to provide better services (+)	No anticipated side-effects. Increase in the statutory vehicle tax is unlikely to substantially reduce demand for vehicles (+)	Calls for improved and transparent financial management (+ –)

Note: (– –) very weak; (–)  rather weak; (+ –)  neutral; (+) strong; (+ +) very strong.

Source: Based on country study.^8^

Table 6 illustrates the quantitative potential for raising revenue of the low-scenario and high-scenario cases (i.e. the combination of all low-scenario settings for each mechanism, or of all high-scenario settings respectively), as well as of the basket of revenue-raising mechanisms that were proposed for further policy consideration (Table 3). The range of estimated additional revenues, as a share of general government expenditure, that could be mobilized from this suggested basket of revenue-raising mechanisms were 0.47–1.62% across the four countries, or 0.52–2.88% for the high-scenario case.^5^^–^^8^

**Table 6:** Illustrations of the estimates of revenues raised under various scenarios

Scenario	First projection year	Projected revenues, US$	Last projection year	Projected revenues, US$	Projected revenues as a share of general government expenditure in the first projection year, %^a^	Projected revenues as a share of GDP, %^a^
**Benin**						
High scenario^b^	2015	36 680 738	2025	75 783 005	1.78	0.42
Proposed for consideration^c^	2015	33 444 464	2025	70 493 807	1.62	0.38
**Mali**						
Low scenario^d^	2016	10 478 967	2024	21 507 687	0.32	0.09
High scenario^d^	2016	40 796 954	2024	86 115 765	1.23	0.34
Proposed for consideration^c^	2016	21 478 015	2024	44 211 372	0.65	0.18
**Mozambique**						
Low scenario (same as Proposed for consideration^c^)	2014	34 557 600	2019	38 267 000	0.47	0.21
High scenario	2014	38 000 008	2019	60 981 700	0.52	0.23
**Togo**						
Low scenario^d^	2014	5 252 688	2024	12 092 065	0.44	0.11
High scenario^d^	2014	34 029 351	2024	77 772 288	2.88	0.74
Proposed for consideration^c^	2014	15 113 063	2024	35 894 263	1.28	0.33

GDP: gross domestic product; US$: international United States dollars.

^a^ Revenue as shares of general government expenditure and GDP were calculated based on 2014 data, using the World Health Organization global health expenditure database.^25^

^b^ In Benin, only a high scenario was calculated.

^c^ Estimates of the basket of mechanisms proposed for policy consideration, listed in Table 3.

^d^ For Mali and Togo, no data were available to project revenues for a new tax on the extractive industries.

Sources: Based on the results of country studies.^5^^–^^8^ Total amounts of revenues per high, low and proposed scenario cases were translated into shares as of general government expenditure and GDP.

## Policy lessons and key issues

The results from both the qualitative and quantitative assessments showed that the proposed new revenue-raising mechanisms could be feasible options for increasing domestic revenues. The estimated additional revenues as a share of general government expenditure from the suggested basket of revenue-raising options are rather small. Nevertheless, even a small increase in revenue is valuable. This finding is in line with the evidence from a recent WHO review that reiterated the importance of increasing fiscal space through new general revenue-raising mechanisms in combination with other strategies to expand the fiscal space for health.^2^

Consideration of various limitations and implementation issues is important. Unavailable or inaccurate data made it impossible to adequately estimate potential revenues for a few mechanisms, particularly for a tax on the extractive industries. There also remains uncertainty about how realistic the assumptions are. These factors affect the strength of the projections. Moreover, it is unlikely that countries would implement the full basket of mechanisms under consideration. Also, the estimates do not consider existing shortcomings in tax administration and collection (including tax evasion, smuggling and the informal economy), which would reduce the estimates of revenues raised.

The stakeholder consultations and interviews revealed that some sectors seemed more attractive than others for the introduction of new revenue-raising mechanisms. This was the case for a new or an increased tax on airplane tickets, telephone calls and (imported) alcoholic beverages in Benin, Mali and Togo. In Mozambique, new taxes on tourism services, alcoholic drinks and the extractive industries and an increased tax on vehicles were considered as possible options. This attractiveness may also relate to the fact that some of these taxes are already in place in other countries in the region and worldwide, and will be paid by a large share of people. For example, Gabon is well known for collecting a tax on the turnover of mobile phone companies.^26^ More than half of the funding for the international drug purchasing facility Unitaid comes from a tax on airline tickets levied by 10 countries^.^^27^ Also, nearly all countries globally already have an excise tax on alcoholic drinks, although few adjust this for inflation.^28^ Moreover, most countries worldwide have a tax on tobacco products and although these taxes are mostly rather low, 106 countries have increased their tobacco excise taxes since 2007, after the Framework Convention for Tobacco Control was ratified.^29^

The country studies further demonstrated that exploring the feasibility of new mechanisms is essential, as it may rule out some of the options that appear promising from the quantitative assessments. For example, country stakeholders considered taxing financial transactions and the extractive industries (in Togo and Mali) as not currently feasible. Also, the studies revealed that a feasibility assessment needs to go beyond national borders to consider the role of sub-regional regulations, such as from the West African Economic and Monetary Community for the three West African countries.^17^

In terms of the process, the country studies confirmed that a wide range of stakeholders and decision-makers need to be included from the very beginning, to create a mutual understanding of the role of new revenue-raising mechanisms, with an ultimate aim of increasing funds for the health sector for progress towards UHC. While finance ministries will lead such discussions, health ministries can contribute in a constructive way to this dialogue. A set of arguments for ministries of health to use in this dialogue have been suggested by other researchers.^30^ The consultation process also allows for raising new considerations for the development of health financing strategies. Moreover, discussions around fiscal space enabled better exchange on health financing with the finance ministry and other ministries and fostered collaborations, as is found by other reseachers.^2^

Finally, it is important to carefully assess whether and if so, when, to bring up the issue of earmarking for health into these discussions in order not to affect the health financing and domestic revenue-raising policy dialogue. International evidence points to the fact that earmarking for health may raise additional resources, but this may be offset by reducing discretionary budget allocations, resulting in little if any overall increased fiscal space for health.^31^^,^^32^ However, from the perspective of finance ministries, tying the messaging and advocacy for a specific tax increase to the health sector may be preferable, as it may increase acceptability by the public.

## Conclusion

Discussions on health financing reforms for UHC are ongoing in the four studied countries and so is the process of reflection about new revenue-raising strategies. As in other countries, these are multi-year processes of political negotiations and decisions on new revenue-raising mechanisms remain to be reported. This type of work, however, can trigger or further inform such policy discussions.

In summary, new revenue-raising mechanisms remain a topical subject, as countries seek to estimate the potential of new revenue-raising mechanisms. With a rising burden of noncommunicable diseases, so-called health taxes (on products high in saturated fat, trans-fatty acids, sugar or salt) receive increasing attention, similar to so-called sin taxes (on tobacco products and alcoholic drinks). However, it needs to be emphasized that the primary rationale of such taxes is to reduce the consumption of products with harmful health consequences. Increasing general government revenues is only a secondary objective.^33^

For future initiatives and studies, there are several key messages. First, whatever the source of additional revenue, in principle such new revenue-raising mechanisms should flow into the general government budget rather than being ring-fenced for a specific sector or disease programme. Second, more attention is needed on how to improve tax collection, which is also part of increasing revenues. Importantly, various publications suggest that improved tax collection is one of the most effective strategies to increase government revenues.^2^^,^^34^^,^^35^ Finally, it is important to remember that new revenue-raising mechanisms represent only one of several strategies to expand fiscal space for health and a combination of strategies is needed. While a health financing strategy highlights the need for additional revenues going to health, overall government revenue-raising must be distinguished from the question of health financing for UHC.
